# Comparing etoricoxib and celecoxib for preemptive analgesia for acute postoperative pain in patients undergoing arthroscopic anterior cruciate ligament reconstruction: a randomized controlled trial

**DOI:** 10.1186/1471-2474-11-246

**Published:** 2010-10-25

**Authors:** Tanarat Boonriong, Boonsin Tangtrakulwanich, Prapakorn Glabglay, Sasikaan Nimmaanrat

**Affiliations:** 1 Department of Orthopaedic Surgery and Physical Medicine, Faculty of Medicine, Prince of Songkla University, Hat Yai, Songkhla, Thailand; 2 Department of Anesthesiology, Faculty of Medicine, Prince of Songkla University, Hat Yai, Songkhla, Thailand

## Abstract

**Background:**

The efficacy of selective cox-2 inhibitors in postoperative pain reduction were usually compared with conventional non-selective conventional NSAIDs or other types of medicine. Previous studies also used selective cox-2 inhibitors as single postoperative dose, in continued mode, or in combination with other modalities. The purpose of this study was to compare analgesic efficacy of single preoperative administration of etoricoxib versus celecoxib for post-operative pain relief after arthroscopic anterior cruciate ligament reconstruction.

**Methods:**

One hundred and two patients diagnosed as anterior cruciate ligament injury were randomized into 3 groups using opaque envelope. Both patients and surgeon were blinded to the allocation. All of the patients were operated by one orthopaedic surgeon under regional anesthesia. Each group was given either etoricoxib 120 mg., celecoxib 400 mg., or placebo 1 hour prior to operative incision. Post-operative pain intensity, time to first dose of analgesic requirement and numbers of analgesic used for pain control and adverse events were recorded periodically to 48 hours after surgery. We analyzed the data according to intention to treat principle.

**Results:**

Among 102 patients, 35 were in etoricoxib, 35 in celecoxib and 32 in placebo group. The mean age of the patients was 30 years and most of the injury came from sports injury. There were no significant differences in all demographic characteristics among groups. The etoricoxib group had significantly less pain intensity than the other two groups at recovery room and up to 8 hours period but no significance difference in all other evaluation point, while celecoxib showed no significantly difference from placebo at any time points. The time to first dose of analgesic medication, amount of analgesic used, patient's satisfaction with pain control and incidence of adverse events were also no significantly difference among three groups.

**Conclusions:**

Etoricoxib is more effective than celecoxib and placebo for using as preemptive analgesia for acute postoperative pain control in patients underwent arthroscopic anterior cruciate ligament reconstruction.

**Trial registration number:**

NCT01017380

## Background

Multimodal or balanced analgesia, using a combination of analgesics throughout the perioperative period to control postoperative pain, has been increasingly popular and well accepted [1,2]. Nonsteroidal anti-inflammatory drugs (NSAIDs) have a significant role in postoperative pain control as they reduce the use of opioids [3-5] which were associated with a variety of postoperative side effects, such as ventilatory depression, drowsiness and sedation, nausea and vomiting, pruritus, urinary retention, ileus and constipation [6,7]. The nonselective NSAIDs inhibit both forms of the cycloxygenase (COX) enzymes. The efficacy of NSAIDs for the treatment of pain is due to the inhibition of the COX-2 enzyme, whereas the inhibition of the COX-1 enzyme may lead to disturbance of normal platelet function and gastrointestinal toxicity [8,9]. Selective COX-2 inhibitors offer significantly less gastrointestinal toxicity and no effects on platelet aggregation [10], therefore are more suitable for perioperative use. A number of studies have shown that these selective COX-2 inhibitors are effective in reducing pain in postoperative period [3-5,7,11-17] and more effective if given both before and after surgery[5,18] . From meta-analysis, a single oral dose of either etoricoxib or celecoxib is an effective means of postoperative pain relief [19,20]. However, we could not find any study of efficacy of single oral dose of these medicines given pre-operatively. Furthermore, the comparisons where usually made between selective cox-2 inhibitors and conventional NSAIDs or other medicines. There has been no head-to-head comparison study between these two novel selective COX-2 inhibitors in terms of postoperative pain reduction. The purposes of this study comparing the efficacy of preoperative administrations of etoricoxib versus celecoxib and placebo for post-operative pain relief after arthroscopic anterior cruciate ligament reconstruction are to evaluate the efficacy of single preoperative dose of selective cox-2 inhibitors and whether there is any superiority among selective cox-2 inhibitors currently available in the market.

## Methods

This study was approved by the Ethic Committee of our Faculty. The patients diagnosed as anterior cruciate ligament injury aged between 15 to 50 years old who scheduled for arthroscopic anterior cruciate ligament reconstruction (ACLR) in Songklanagarind hospital during January 2008-January 2009 was included in the study. We excluded the patients who had known allergy, sensitivity or contra-indications to opioids or NSAIDs, having a history of dyspepsia, peptic ulcer or abnormal bleeding, coronary and peripheral arterial diseases as well as allergy to sulfonamide group. The patients who had used NSAIDs, opioids, salicylate within 7 days of the operation were also excluded. The patients were randomized into 3 groups; etoricoxib,celecoxib and placebo using random table containing in the opaque envelope. In etoricoxib group, 120 mg of etoricoxib was orally given. In celecoxib group, 400 mg was given 1 hour before the incision as same as in the controlled group. Both the surgeon and the assessors were blinded to the result of allocation. As a currently common treatment protocol in our country where the hospital cost is inexpensive and for the purpose of direct pain observation, all patients were admitted a night before surgery and discharged at 48 hours post-operatively. The operations were performed under spinal anesthesia using 0.5% hyperbaric bupivacaine without additional intrathecal opioid. The arthroscopic anterior cruciate ligament reconstructions were performed by the principle investigator using autograft bone-patellar tendon-bone. The operative time were recorded. At the end of the operation, all the remaining intra-articular fluid was squeezed out, a vacuum drain was placed intra-articularly and the operative wound was closed before the tourniquet was released. The total drain amount was recorded at 48 hours before removal.

All subjects were treated in routine fashion in the recovery room. They were kept until they recovered from the spinal anesthesia and satisfied with recovery room scoring system. The operated knees were locked at in full extension with hinge knee brace for 48 hours and allowed for 0-90 degrees motion at 48 hours when the drain had been removed and wound had been dressing changed. During the post-operative period, the patients were asked to quantify their pain using a Verbal Analog Pain Scale (VbAPS) of 0-100 mm. where 0 represents no pain and 100 mm for unbearable pain. The first pain evaluation was made just before they left the recovery room and then repeated at 4, 8,12, 16, 20, 24, 30, 36, 42 and 48 hours postoperatively. The post-operative pain medications allowed were oral paracetamol 1000 mg taken as needed every 6 hours and/or intravenous fentanyl 1 microgram per kilogram taken every 3 hours as requested by patients. The time to first use of each analgesic medication was recorded. The total amount of both medications was recorded at 48 hours. The patients were also asked to grade their satisfaction with pain control at 48 hours using a Verbal Analog Pain Scale (VbAPS) of 0-100 mm, where 0 represent dissatisfy and 100 mm for most satisfy. The primary outcome of this study was a comparison of the postoperative pain levels in the three groups, while the secondary outcomes were the time to first analgesic analgesics, total amount of analgesics used, the amount of drain output (as represent blood loss) and patients' satisfaction with their pain control. The vital signs were recorded regularly. All adverse drug reactions and side effects were also recorded.

### Statistical analysis

The sample size required for this study was 32 patients in each arm. The calculation relied on the primary outcome; post-operative pain intensity, which from previous study found the standard deviations of celecoxib and etoricoxib to be 0.2 and 0.3, respectively. The sample size was based on a 2-sided test with 80% power and a significant level of 0.05. All statistical calculation was performed using STATA version 9.0.We analyzed the data based on intention-to-treat principle. Either chi-square or Fisher's exact test was used to analyze categorical outcomes. Differences among 3 groups of continuous variables were analyzed by analysis of variance (ANOVA). Post-hoc analysis was performed with Bonferroni test.

## Results

**Figure 1:**
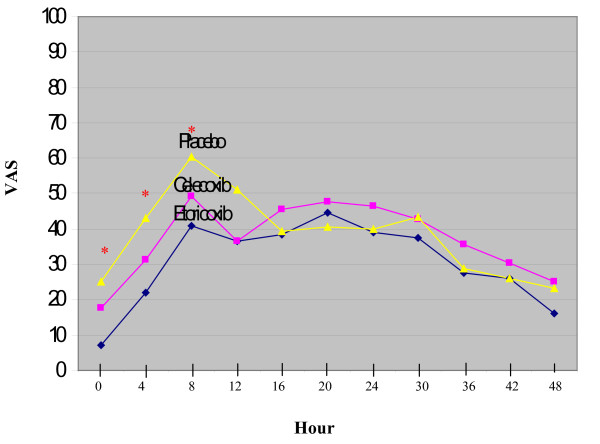
**Pain Intensity among each group during 48 hour after surgery**.

Among 102 patients, 35 were in etoricoxib, 35 in celecoxib and 32 in placebo group. The mean age of the patients was 30 years and most of the injury came from sports injury. There were no significant differences among groups in all demographic characteristics. The duration of injury, associated injuries, concomitant surgeries, mean operative and tourniquet time, and mean drain output were also no differences among groups (Table 1). There were no differences among groups for the time to first dose of analgesic medication, amount of paracetamol and fentanyl used, and patient satisfaction with pain control (Table 2). Although there were significantly higher reports of constipation and fever in placebo group, there was no significant difference in the numbers of adverse event among the three groups (Table 3). The etoricoxib group had significantly less pain intensity than the other two groups at recovery room and up to 8 hours period but no significance difference in all other evaluation point, while celecoxib showed no significantly difference from placebo at any time point (Figure 1).

**Table 1:** Patient characteristics among groups

Characteristics	Etoricoxib	Celecoxib	Placebo	P-value
· Number	35	35	32	
· Sex: Male (%)	33 (94.29)	31 (88.57)	28 (87.50)	0.375
· Mean duration of injury (SD)	18.22 (15.93)	28.31 (44.87)	35.41 (49.23)	0.477
· Associated injury				0.234
None	9 (25.71)	7 (20.00)	9 (28.13)	
Medial meniscus	16 (45.71)	17 (48.57)	10 (31.25)	
Lateral meniscus	5 (14.30)	7 (20.00)	6 (18.75)	
Both meniscus	2 (5.71)	1 (2.86)	7 (21.88)	
Articular injury	2 (5.71)	2 (5.71)	0 (0)	
Both meniscus and articular injury	1 (2.86)	1 (2.86)	0 (0)	
· Concomitant surgery				0.75
None	13 (37.15)	13 (37.14)	13 (40.63)	
Medial menisectomy	13 (37.15)	11 (31.43)	10 (31.25)	
Lateral menisectomy	3 (5.87)	5 (14.29)	6 (18.75)	
Both medial and lateral menisectomy	2 (5.71)	0 (0)	2 (6.06)	
Cartilage debridement	2 (5.71)	5 (14.29)	2 (6.25)	
· Mean pain score before surgery (SD)	11.17 (17.76)	14.0 (21.58)	15.62 (26.39)	0.721
· Mean operative time (SD)	52.50 (10.76)	60.73 (14.20)	60.45 (20.30)	0.223
· Mean tourniquet time (SD)	56.08 (9.69)	63.25 (12.84)	63.90 (19.33)	0.089
· Mean drain output at 24 hr./ml (SD)	170.77 (183.68)	218.94(231.48)	143.28(106.40)	0.663

**Table 2:** Medication usages and patient satisfaction among groups

Variables	Etoricoxib	Celecoxib	Placebo	P-value
· Mean number of rescue medication (SD)				
- Paracetamol (Tabs.500 mg.)	3.05 (2.44)	3.50 (2.83)	4.65 (3.49)	0.648
- Amount of fentanyl use (micrograms)	114.64 (89.99)	112.8 (84.43)	171.43 (129.56)	0.222
· Time to first dose of fentanyl (hr)	5.89 (5.67)	5.83 (10.22)	5 (5.85)	0.55
· Mean pain satisfaction score	78.14 (14.04)	80.68 (16.86)	72.22 (18.36)	0.167

**Table 3:** Adverse events among groups

Adverse events	Etoricoxib	Celecoxib	Placebo	P-value
**· Gastrointestinal**				
Dyspepsia	1 (2.85)	0 (0)	2 (6.25)	0.436
Flatulence	0 (0)	0 (0)	1 (3.13)	0.205
Nausea	1 (2.85)	0 (0)	0 (0)	0.233
Vomiting	0 (0)	0 (0)	2 (6.25)	0.07
Constipation	0 (0)	0 (0)	3 (9.38)	0.025*
**· Neurological**				
Dizziness	3 (8.57)	2 (5.71)	4 (12.50)	0.59
Headache	0 (0)	1 (2.85)	2 (6.25)	0.133
**· Cardiovascular**				
Tachycardia	2 (5.71)	0 (0)	3 (9.38)	0.52
Hypertension	3 (8.57)	2 (5.71)	5 (15.63)	0.35
**· Renal**				
Oliguria	0 (0)	0 (0)	1 (3.13)	0.205
**· Other**				
Fever	2 (5.71)	12(34.29)	11 (34.38)	0.005*

* Statistically significant among groups

## Discussion

Postoperative pain following arthroscopic ACL reconstruction could be perceived by the patients immediately in the recovery room as they were recovering from spinal anesthesia, rose to peak intensities around 8 hours and slowly decreased to acceptable level below 40 after 36 hours postoperatively. We found that only etoricoxib, not celecoxib, was effective for use as preemptive analgesia in patients undergoing arthroscopic ACL reconstruction. However, the time to first dose of analgesic medication, total amount of analgesics used, and patient's satisfaction with pain control were also not significantly different among the three groups.

Preemptive analgesia is a new strategy of postoperative pain management. The key concept is to prevent the altered sensory processing from surgical process. There are a number of medications being tested for this strategy, including opioids, anesthetic drugs and NSAIDs with conflicting results [3-5,7,21,22]. Although there have been some studies evaluating the efficacy of NSAIDs for preemptive analgesia, our study is the first head-to-head study of using COX-2 inhibitor NSAIDs for preemptive analgesia for major orthopaedic surgery. Our results found that only etoricoxib was efficacious for use as preemptive analgesia after major orthopaedic surgery. Efficacy of etoricoxib over celecoxib and the placebo may explained by the better or stronger analgesic efficacy of this drug. Other study had also demonstrated the efficacy of etoricoxib for perioperative pain control. Rasmussen et al use etoricoxib 120 mg/day from day 1 to day 7 postoperatively in the patient undergoing arthroplasty [17]. They found that etoricoxib provided analgesia that was similar to controlled-release naproxen sodium on day 1 and superior to placebo with reduced supplement opioid used over 7 days [17].

The only head-to-head comparison between etoricoxib and celecoxib was in study of Bingham III, et al. [23] who compared the efficacy of etoricoxib 30 mg with the generally maximum recommended dose of celecoxib, 200 mg, in the treatment of osteoarthritis (OA) in two identically designed studies. They concluded that etoricoxib 30 mg per day was at least as effective as celecoxib 200 mg per day and had similar safety in the treatment of knee and hip OA; both were superior to placebo [23]. Our study reached a contrast conclusion that only etoricoxib, not celecoxib, is effective for reducing pain intensity at the recovery room up to 8 hours postoperatively.

## Conclusions

Etoricoxib is more effective than celecoxib and placebo for using as preemptive analgesia for acute postoperative pain control in patients underwent arthroscopic anterior cruciate ligament reconstruction.

## Pre-publication history

The pre-publication history for this paper can be accessed here:

http://www.biomedcentral.com/1471-2474/11/246/prepub
